# Effects of Steel Slag Powder Content and Curing Condition on the Performance of Alkali-Activated Materials Based UHPC Matrix

**DOI:** 10.3390/ma16103875

**Published:** 2023-05-21

**Authors:** Kangyi Shi, Hongyang Deng, Jinxuan Hu, Junqi Zhou, Xinhua Cai, Zhiwei Liu

**Affiliations:** 1 School of Civil Engineering and Architecture, Wuhan Polytechnic University, Wuhan 430072, China; shikangyisky@126.com (K.S.); hujinxuan@whpu.edu.cn (J.H.); wlmm1128@yeah.com (J.Z.); tonyliuzhiwei@whpu.edu.cn (Z.L.); 2 State Key Laboratory of Water Resources and Hydropower Engineering Science, Wuhan University, Wuhan 430072, China; caixinhua@whu.edu.cn

**Keywords:** ultra-high-performance concrete, steel slag powder, alkali activated materials, curing condition, microstructure

## Abstract

The accumulation of steel slag and other industrial solid wastes has caused serious environmental pollution and resource waste, and the resource utilization of steel slag is imminent. In this paper, alkali-activated ultra-high-performance concrete (AAM-UHPC) was prepared by replacing ground granulated blast furnace slag (GGBFS) powder with different proportions of steel slag powder, and its workability, mechanical properties, curing condition, microstructure, and pore structure were investigated. The results illustrate that the incorporation of steel slag powder can significantly delay the setting time and improve the flowability of AAM-UHPC, making it possible for engineering applications. The mechanical properties of AAM-UHPC showed a tendency to increase and then decrease with the increase in steel slag dosing and reached their best performance at a 30% dosage of steel slag. The maximum compressive strength and flexural strength are 157.1 MPa and 16.32 Mpa, respectively. High-temperature steam or hot water curing at an early age was beneficial to the strength development of AAM-UHPC, but continuous high-temperature, hot, and humid curing would lead to strength inversion. When the dosage of steel slag is 30%, the average pore diameter of the matrix is only 8.43 nm, and the appropriate steel slag dosage can reduce the heat of hydration and refine the pore size distribution, making the matrix denser.

## 1. Introduction

The main characteristics of ultra-high performance concrete (UHPC) are high compactness, high strength, and high durability, which are obtained by using a low water-cement ratio and batching with reference to the particle dense packing theory in the production process [1,2]. The unit cubic cement consumption of UHPC is four times higher than that of ordinary concrete, reaching 800~1000 kg/m^3^ [3], while each 1 t of ordinary Portland cement (OPC) produced emits 0.82 t of CO_2_ into the atmosphere [4]. The high cement consumption makes UHPC costly and also has a negative impact on the environment and the heat of hydration [5,6,7], so with the arrival of the carbon peak in the construction industry, more and more researchers are focusing on using new materials and technologies to reduce the cement consumption, carbon emission, and energy consumption of UHPC while ensuring its excellent performance [8].

China’s annual industrial solid waste is about 3.6 billion tonnes, with a historical stockpile of over 60 billion tonnes, covering an area of over 20,000 square kilometres [9]. Among them, nearly 3 billion tonnes of steel slag are not effectively used [10], and the damage to the environment cannot be ignored. Steel slag is an industrial solid waste produced during the steel making process to remove impurities from the steel. Although steel slag is utilized in agriculture, environmental protection and construction [11], it is utilized in less than 30% of low value-added applications [12,13] such as fillers for basic construction or asphalt mixes due to its poor water hardening activity. Alkali activated materials (AAM) typically use silica-alumina raw materials such as ground granulated blast furnace slag (GGBFS) [14,15] and fly ash [16,17] as precursors, and alkali water glass [18] and hydroxide [19] as activators. AAM is considered as one of the alternatives to OPC [20,21] because of lower energy consumption and CO2 emission, and it can make full use of industrial solid waste and solve the environmental problems caused by solid waste dumping. Zeng Lu et al. [22] used water glass as an activator and steel slag and GGBFS as cementitious materials to produce all-solid waste aerated concrete with better performance. Wang X et al. [23] prepared environmentally friendly steel slag-based cementitious materials from industrial solid wastes such as steel slag and calcium carbide slag, and their study showed that steel slag-based all-solid waste cementitious materials with high blending of steel slag are safe for the environment.

In the preparation of ecological UHPC [24,25,26,27,28], there have been some reports on the use of solid waste-based admixtures such as steel slag powder, GGBFS powder and fly ash to replace part of the OPC, but there are few studies on the use of solid waste-based raw materials to replace all the silicate cement in the preparation of UHPC. Chen et al. [29] investigated the effect of alkaline activator dosage on the performance of alkali activated GGBFS concrete, which reached more than 100 MPa after 28 days of curing, and the mechanical strength of the specimens was close to that of UHPC. Choi J I et al. [30] used polyethylene fibers in combination with alkali activated GGBFS to prepare ultra-high toughness concrete with a tensile strain capacity of 7.5%. Cai R et al. [31,32] used a variety of activators and GGBFS to produce ultra-high strength concrete with a 28 d compressive strength of 120 MPa or more.

The prerequisite for a homogeneous UHPC mix is good workability, which requires UHPC mixes with excellent flowability, cohesiveness and water retention. The setting time is also one of the important workability properties of concrete mixes, which determines the time window for mixing, transporting and placing the mixes. One of the disadvantages of applying GGBFS as a precursor in alkali activated cementitious materials is its rapid setting time [33,34,35,36], especially when mixed with more activator to obtain higher mechanical properties [37], its setting is extremely rapid, making it extremely limited in practical engineering applications [38]. You N et al. [39] found that the amount of steel slag at 50% can significantly delay the setting time of alkali activated mortar and improve the flowability.

In this study, based on the concept of "zero-waste city" and environmental sustainability, the industrial solid wastes such as steel slag and GGBFS were used as precursors. By adjusting the ratio of raw materials to optimise the particle buildup. Na_2_O-9SiO_2_ and K_2_CO_3_ were used as activators to improve the active reaction. The AAM-UHPC was prepared with excellent performance, and the effects of different dosages of steel slag and curing conditions on the performance of AAM-UHPC were investigated through comparative tests.

## 2. Materials and Methods

### 2.1. Raw Materials

Precursors:Steel slag(SS) powder was supplied by Huailong New Building Material Co. (Jiangsu, China). Ground granulated blast furnace slag powder was S95 GGBFS powder produced by Hengle New Building Materials Co. (Zhangjiagang, China). The semi-densified silica fume (SF) was produced by Huashen Smart Technology Co., Ltd. (Wuhan, China) with a specific surface area of 19.915 g/cm^3^. In order to avoid test errors caused by large particles agglomerated in the silica fume, it was screened with a 0.015 mm square hole sieve before use. The chemical compositions of the raw materials used in this research are provided by manufacturers (Table 1).

Na_2_O-9SiO_2_ and K_2_CO_3_ of analytical grade were adopted to prepare the alkaline activator solutions. Quartz sand with particle sizes ranging from 0 to 0.5 mm, 0.5 to 1 mm, and 1 to 2 mm was selected as fine aggregate, with a mass ratio of 1:1.75:2.25 and a ratio of 1.2 for the sand and solid materials.

### 2.2. Mix Proportions and Specimens Preparations

In order to study the effects of steel slag admixture and curing conditions on the performance of AAM-UHPC, the mixing ratio design was optimized according to the modified Andreasen & Andersen (MA&A) model. First, the target curve was determined according to the MA&A model as shown in Equation (1), and then the particle size distribution curve was made close to the target curve by adjusting the proportion of each solid material, and the detailed mixture proportions are provided in Table 2.

AAM-UHPC is prepared by first dissolving Na_2_O-9SiO_2_ and K_2_CO_3_ in water while mixing the solid material and quartz sand well, then adding the liquid phase slowly into the solid mixture, followed by slow stirring for 90 s and continuously fast stirring quickly for 60 s to make AAM-UHPC matrix, and finally demolding after 24 h:

(1) $$P(D)=\frac{D^{q}-D_{\min}^{q}}{D_{\max}^{q}-D_{\min}^{q}}$$

where *D* signifies the particle size (μm); *P*(*D*) represents the percentage content of particles with particle size less than *D*; *D*_max_ represents the maximum particle size (μm); *D*_min_ represents the minimum particle size (μm); and *q* indicates the distribution modulus, which is taken as 0.23 [40].

### 2.3. Curing Condition

A total of three types of curing conditions were used in this study. Standard curing consisted of placing the specimens in a constant temperature and humidity maintenance chamber with the temperature set at 20 ± 1 °C and the relative humidity ≥ 90% until the corresponding test age.

Steam curing was performed in an accelerated concrete curing chamber by placing the specimens in the upper steam layer of the curing chamber without touching the lower water storage layer, and the heating rate was set at 10 °C/h until it reached 60 °C, and the cooling rate was set at 15 °C/h until it dropped to room temperature. In this study, the steam curing was continued for 3 d and 7 d. At the end of 7 d, the specimens were taken out and put into the standard curing box to continue the standard curing for 28 d. The hot water curing was performed by placing the specimens in the water storage layer at the bottom of the accelerated concrete curing chamber, and the temperature rise and fall rates were the same as those of the steam curing.

### 2.4. Testing Methods

The setting time was determined using a Vicat apparatus with reference to the Test Method for Water Consumption, Setting Time, and Settlement of Cement Standard Consistency (GB/T 1346-2011) [41]. Since the setting time of activated cementitious materials is fast, one test was conducted immediately after the paste was poured into the metal mold, every 2 min in the early stage and every 1 min in the late stage.

A jump table was used to measure the flow of AAM-UHPC according to the Method for Determination of Cementitious Sand Flow (GB/T 2419-2005) [42].

The compressive strength test was conducted according to the Standard Test Method for Compressive Strength of Cement Mortar (ASTM C109-12) [43], and the specimens prepared for the compressive strength test were 50 mm × 50 mm × 50 mm. The flexural strength test of the specimens was conducted according to the Test Method for Strength of Cement Mortar (ISO Method) (GB/T 17671-1999) [44], and the specimens were 40 mm × 40 mm × 160 mm. The average value of three specimens in each group was taken as the final test value.

XRD analysis of the powder samples was performed using a D8 Advance (Bruker, Billerica, MA, USA) with Cu-Kα (λ = 1.54056 Å) scanned in the degree of 5–70°, counting time 120 s.

A Gemini SEM 300 (ZEISS, Oberkochen, Germany) scanning electron microscope was used for the observations of the specimens, and core samples were taken from 7 groups of specimens cured for 3 d. The pore structure was analyzed using a V9620 (Micromeritics, Norcross, GA, USA) high-performance mercury piezometer with a maximum feed force of 60,000 psi. All specimens were dried in an oven at 60 °C for 8 h prior to testing.

## 3. Results and Discussion

### 3.1. Influence of Steel Slag Powder Dosage on Workability

**Figure 1:**
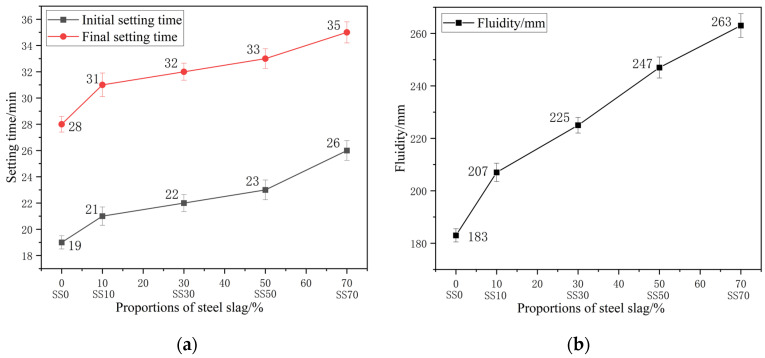
Effect of different steel slag dosages on the workability of AAM-UHPC: (**a**) Setting time of different steel slag dosages; (**b**) Fluidity of different steel slag dosages.

Figure 1a shows the effect of steel slag powder dosage on the setting time of AAM paste. The results indicate that the setting time of AAM paste gradually increases with the increase in steel slag powder dosage. The main reason is that steel slag powder, as a relatively more inert powder particle than GGBFS powder, reacts much less than GGBFS powder in the early stage and reduces the production of reaction products [45], which can delay the setting of the paste. From the SS0 group to the SS10 group, the initial and final setting times increased by 10.5% and 10.7%, respectively, when the dosage of steel slag was increased to 10%, while from the SS50 group to the SS70 group, the initial and final setting times only increased by 13% and 6%, respectively, when the amount of steel slag increased by 20%. In the case of a lower steel slag powder dosage, the continued addition of steel slag powder has a more outstanding contribution to delay the paste coagulation.

The flowability of different steel slag dosages is shown in Figure 1b. The results showed a positive relationship between the steel slag dosage and the flowability of AAM-UHPC. The adsorption of water onto the different materials is different [46], and the water requirement of steel slag powder is less than that of GGBFS powder [47,48,49]. As the amount of steel slag powder increases, the mass proportion of GGBFS powder also decreases, the free water in the liquid phase relatively increases [50], the C-S-H gel generated in the paste in the short term also decreases, and at the same time the viscosity decreases, the flowability of the mix is improved.

### 3.2. Influence of Steel Slag Powder on Mechanical Properties

**Figure 2:**
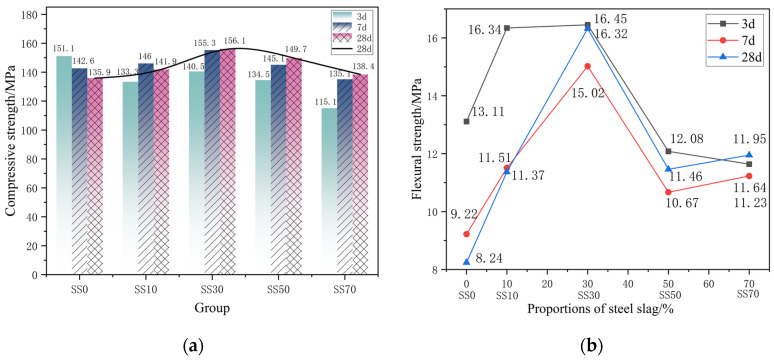
Effect of different steel slag dosages on the mechanical properties of AAM-UHPC: (**a**) Compressive strength of steel slag with different dosages; (**b**) Flexural strength of steel slag with different dosages.

Figure 2 shows the compressive and flexural strengths of AAM-UHPC prepared with different steel slag dosages for 3 d, 7 d, and 28 d. The results showed that the 28 d compressive strength of AAM-UHPC showed a pattern of increasing and then decreasing with the increase in steel slag dosage. In terms of later strength, at 28 d, the compressive and flexural strengths of the SS30 group were the highest, reaching 156.1 MPa and 15.08 Mpa, respectively. From Figure 2a, compared with the SS0 group without steel slag, it can be found that the compressive strength of the SS30 group increased by 14.8%, reaching 156.1 MPa.

The early strength and late strength of the concrete were improved by the binary composite activation of steel slag powder and GGBFS powder. At the same time, the incorporation of steel slag can significantly improve workability, which shows a good superimposed effect [51]. The compressive strength of the SS30 group is 12.7% higher than that of the SS70 group with a high amount of steel slag, and the excessive amount of steel slag leads to a relative reduction of active components in the system, which in turn leads to a decrease in mechanical properties.

From Figure 2b, it can be found that the flexural strength of AAM-UHPC first increases and then decreases with the increase in steel slag dosage, reaching 16.45 MPa in the SS30 group. The flexural strength of the SS0 group at 28 d decreased by 37.14% compared with that at 3 d, while that of the SS30 group decreased by only 0.7% and that of the SS70 group increased by 2.6%. It can be concluded that although the active components of GGBFS in the early steam curing are almost depleted in the reaction, the better crystallized C_2_S and other minerals in the steel slag powder will continue to hydrate at a later stage. The generated hydration products fill the pores and provide a guarantee for the growth of mechanical properties at a later stage, and the degree of inversion of flexural strength gradually decreases with the increase in steel slag admixture.

At each age, the compressive and flexural strengths of the SS10 group and SS30 group met the criteria of RPC100 grade in the specification Reactive Powder Concrete (GB/T 31387-2015) [52]. Based on the workability and mechanical properties of each group, the SS30 group was selected for the production of AAM-UHPC.

### 3.3. Influence of Curing Condition on the Mechanical Properties

At present, in order to maximize the use of the cementing activity of steel slag, the activation methods commonly used in the industry include mechanical activation, chemical activation, and high-temperature activation. Studies have shown [53] that Si-O bonds and Al-O bonds in steel slag are more likely to break at high temperatures, and high temperatures are conducive to glass depolymerization, which can accelerate the reaction process of steel slag under chemical activation. Both steam and hot water curing belong to the category of high-temperature activation in this paper.

**Figure 3:**
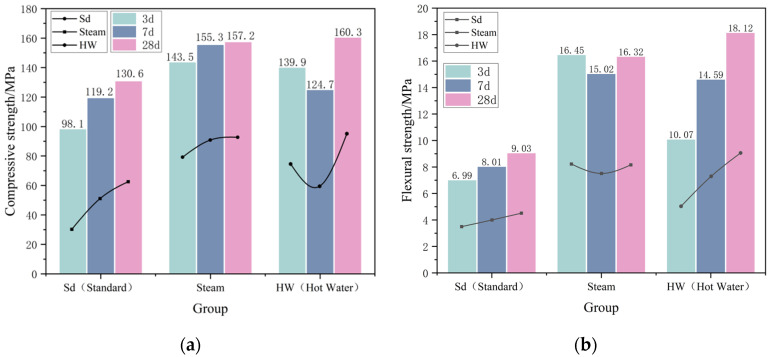
Effect of different curing conditions on the mechanical properties of AAM-UHPC: (**a**) Compressive strength of different curing conditions (Group SS30); (**b**) Flexural strength of different curing conditions (Group SS30).

The mechanical strengths of AAM-UHPC at 3 d, 7 d, and 28 d under different curing conditions are shown in Figure 3. The results showed that the 3 d steam and hot water curing significantly improved the mechanical properties, but the long-term hot and humid curing was detrimental to the mechanical property development of AAM-UHPC. The compressive strengths at 3 d for steam and hot water curing were 46.2% and 42.6% higher than those in the standard curing case, respectively. The compressive strength of the steam-cured specimens at 7 d was 8.2% higher than that at 3 d, while that of the hot water-cured specimens was 10.8% lower. Such a reverse shrinkage of mechanical properties was also observed in the flexural strength of the steam-cured specimens at 7 d, which was reduced by 8.7%, but the mechanical properties recovered with subsequent standard curing up to 28 d.

From Figure 3, it can be seen that the late strength increase in steam-cured specimens is extremely small, with the compressive strength of the subsequent curing to 28 d increasing by only 1.2% over that of the steam-cured 7d, and this phenomenon is consistent with that of high-strength concrete. The reason is that the early high temperature makes the reaction proceed rapidly, and the hydration products are extremely dense, so it is difficult for pore water to penetrate from the surface of the specimen to the internal unhydrated particles. The late hydration conditions are obstructed, leading to a serious internal water shortage, which will produce a large self-drying shrinkage, resulting in internal stresses and affecting the late strength growth of concrete [54,55].

The reason for the increase in mechanical strength of AAM-UHPC at the age of 28 d under three curing conditions in this paper is that the C_2_S in the steel slag continues to hydrate at a later stage, and the resulting gel continues to fill the matrix pores to ensure the strength development. Comparing the different curing conditions, AAM-UHPC is suitable for the curing conditions of steam curing for 3 d in the first stage and standard curing up to 28 d in the later stage.

### 3.4. Analysis of Microstructure and Phase Composition

**Figure 4:**
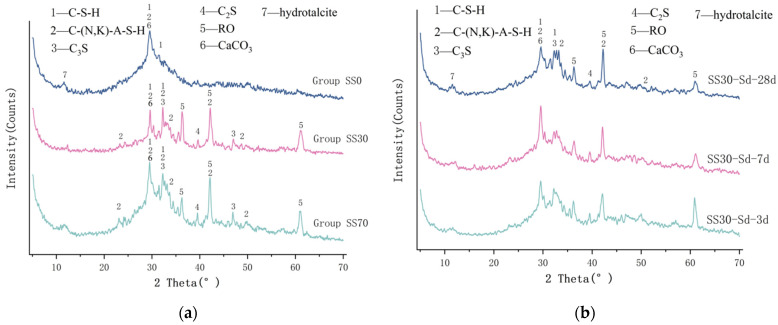
XRD patterns of AAM-UHPC: (**a**) Different steel slag dosage; (**b**) Different curing conditions.

As shown in Figure 4a, the intensity of the diffraction peak of the crystal increases gradually. In the XRD pattern of the SS0 group without steel slag, there are glassy amorphous diffraction broad humps, and the main reaction products are C-(N,K)-A-S-H gels and hydrotalcite. The hydration products in the SS30 and SS70 groups are basically the same, which are mainly C-S-H gel and C-(N,K)-A-S-H gel, the inert RO phase (continuous solid solution composed of divalent metal oxides), and CaCO_3_ generated by carbonation. Despite 3 d steam curing, the peak of the original mineral C_2_S in steel slag still exists. As can be seen from Figure 4b, with the development of standard curing, the peak of C_2_S gradually decreases and gel products increase. Later hydration may be dominated by C_2_S, producing more C-S-H. This explains why the mechanical strength of the group mixed with steel slag in Figure 2a continued to increase in the later period. Ca(OH)_2_ and AFt were not found in the XRD pattern; the reason was that the hydration reactions of steel slag and GGBFS promoted each other, leading to the consumption of Ca(OH)_2_ in the paste and the formation of C-S-H gel.

AAM-UHPC is mainly composed of hydration products generated by the reaction of steel slag powder and GGBFS powder interwoven to form a spatial network. The unhydrated powder and inert components in steel slag play a filling effect, and they are closely packed together to form a dense matrix.

**Figure 5:**
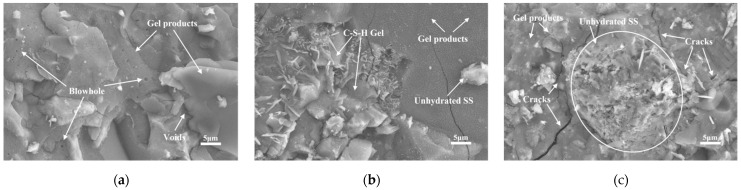
Microstructure of 3D AAM-UHPC with different dosages of steel slag: (**a**) Group SS0-3d; (**b**) Group SS30-3d; (**c**) Group SS70-3d.

The SEM images at 3 d of steaming with different steel slag admixtures are shown in Figure 5. From Figure 5a, it can be observed that the matrix is flat, dense, and smooth, and there are closed pores exposed on the surface due to the breakage of the test block when taking samples, which were originally inside the matrix. The matrix in Figure 5b is more dense, almost no holes can be observed, and some unhydrated steel slag powder particles exist on the surface, which will continue to react with the alkaline material at a later stage. Due to the higher steel slag dosage, the matrix in Figure 5c is relatively more porous, with the presence of unhydrated steel slag particles in the middle of the figure. Amorphous C-S-H gels grow from the surface and near the steel slag particles, and cracks develop along weak interfaces near the steel slag particles during crushing.

**Figure 6:**
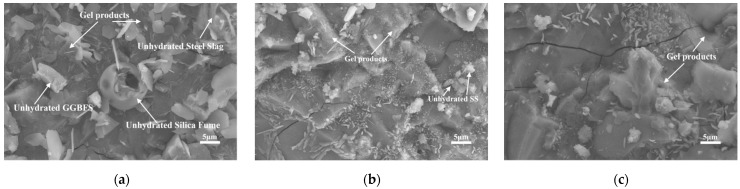
Microstructure of 3D AAM-UHPC under different curing conditions: (**a**) Group SS30-Sd (Standard)-3d; (**b**) Group SS30 (Steam)-3d; (**c**) Group SS30-HW (Hot Water)-3d.

Figure 6 shows the SEM images of the SS30 group at 3 d under different curing conditions. From Figure 6a, it can be found that C-S-H gels were formed in large quantities in the samples with standard curing, and some unhydrated GGBFS powder and steel slag powder particles, hexagonal sheet-like hydrotalcite, and spherical silica fume in the center of the image were not fully reacted. In Figure 6b,c, it can be observed that the matrix is compact and flat under high temperature curing. The original amorphous C-S-H gel forms a monolithic plate, the unhydrated particles are smaller in size, and hydrotalcite exists in the form of single crystals and fills the gaps between C-S-H and C-(N,K)-A-S-H gels, which not only improves the pore structure of the paste but also increases the density of the matrix, thus improving the early mechanical properties of AAM-UHPC.

### 3.5. Analysis of Pore Structure

The AAM-UHPC cured for 3 d was tested for pressures ranging from 0 to 60,000 psi; the porosity of the SS0, SS30, and SS70 groups was 5.07%, 6.71%, and 4.98%, respectively, and the porosity of the S30-St and S30-HW groups was 10.66% and 5.35%, respectively. The porosity and pore size distributions of each group are listed in Table 3.

As shown in Table 3, the average pore sizes of the SS0 and SS70 groups for 3 d of steam conditioning were 47.21 nm and 48.35 nm, but only 8.43 nm for the SS30 group. Combined with their pore size distributions, it can be found that the pore size of the SS30 group is mainly extremely fine gel pores.

The reasons for the excellent pore size distribution of the SS30 group are as follows: on the one hand, compared with the SS0 group, after mixing the correct amount of steel slag powder, it slows down the setting speed of the system, which can make the air bubbles brought in during mixing discharge in time, and the particle size of steel slag powder can fill the particle size range of the break in mineral powder particle grading, thus improving the continuity of the overall gelling material grading; On the other hand, compared with the SS70 group, the hydration release of large amounts of Ca^2+^ from steel slag with a high dosage will inhibit the hydration of GGBFS [56], and the continuous hydration degree of the GGBFS and steel slag is reduced, which also reduces the output of the hydration product gel and increases the porosity of the hardened matrix, especially the harmful void, leading to a reduction of mechanical properties. The particle stacking effect of the SS30 group is more compact, with hydration products filling the pores between powder particles. When the appropriate amount of steel slag is added, the hydration of steel slag and slag promotes each other, which also explains the source of its excellent mechanical properties.

**Figure 7:**
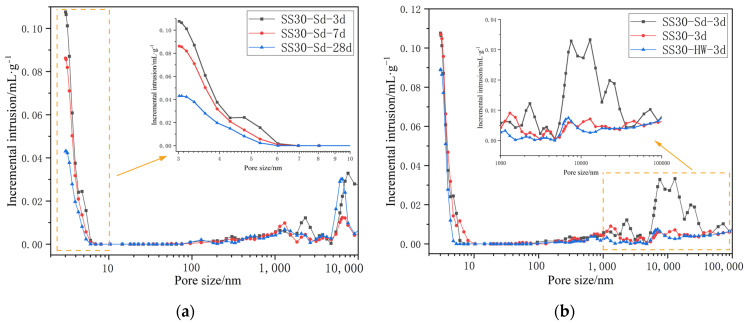
Pore size distribution of AAM-UHPC: (**a**) Different standard curing time; (**b**) Different curing conditions.

As shown in Figure 7, the pore size of AAM-UHPC in all groups is dominated by harmless pores below 20 nm. From Figure 7a, it can be seen that the gel products continue to be generated as the curing progresses, the total porosity decreases, and the pore size distribution shifts toward smaller pore sizes, which also proves that there is a synergistic effect between steel slag powder and GGBFS powder [57]; both the gel formed by GGBFS powder in the early reaction to provide the initial strength and the continued hydration by steel slag powder promote the densification of the structure and ensure the development of the later strength. From Figure 7b, it can be seen that the pore sizes of specimens under both steam and hot water curing conditions are dominated by harmless pores below 20 nm and less harmful pores from 20 to 100 nm, while the standard maintenance specimens are dominated by more harmful pores >200 nm, indicating that the high temperature curing at early ages is beneficial for the reaction of each solid waste component to proceed in an alkaline environment. The lower porosity is an important reason for AAM-UHPC to maintain better mechanical properties.

## 4. Conclusions

Blending with an appropriate amount of steel slag powder can improve the performance of AAM-UHPC. When the amount reaches 30%, AAM-UHPC matrix can reach the highest compressive strength of 156.1 MPa and the highest flexural strength of 16.32 MPa, and it has both better working properties and mechanical properties.

Steel slag powder significantly improves the workability of AAM-UHPC, particularly in terms of delaying the setting time and improving the flowability, thus making AAM-UHPC more suitable for engineering applications.

Steam or hot water curing at an early age can promote the densification of the matrix structure, reduce porosity, and increase the volume of harmless pores, which is beneficial to strength development, but long-term hot and humid curing is detrimental, AAM-UHPC containing steel slag is suitable for early 3 d steam curing combined with the subsequent standard curing.

GGBFS provides the early strength, steel slag ensures the later strength, and their synergistic effect promotes the development of the mechanical properties of AAM-UHPC.

The results of the present study indicate the need for further research aimed at the suitability of the matrix with fibers.

## Figures and Tables

**Table 1:** Chemical compositions (wt.%) and loss on ignition (LOI) of the precursors.

	CaO	SiO_2_	Al_2_O_3_	MgO	Fe_x_O_y_	MnO	P_2_O_5_	TiO_2_	f-CaO	LOI
Steel Slag	33.26	14.52	2.9	5.68	26.53	4.35	2.41	-	-	10.62
GGBFS	37.41	30.92	15.74	8.72	0.3	-	-	2.15	0.04	4.72
Silica Fume	0.38	97.51	0.16	0.88	-	-	0.25	-	-	0.98

**Table 2:** Mixture proportions (wt.%) of AAM-UHPC (mass fraction).

Group	Steel Slag	GGBFS	Silica Fume	Water	Activator		Curing Condition
					Na_2_O·9SiO_2_	K_2_CO_3_	
SS0	0	90	10	0.23	9.5	6.7	Steam
SS10	10	80					
SS30	30	60					
SS50	50	40					
SS70	70	20					
SS30-Sd	30	60					Standard
SS30-HW	30	60					Hot water

**Table 3:** Different groups of AAM-UHPC porosity and pore size distribution.

Group	Porosity/%	Average Pore Size/nm	Pore Size Distribution/%			
			<20 nm	20~100 nm	100~200 nm	>200 nm
SS0-3d	5.07	47.21	60.59	6.50	8.94	23.98
SS30-3d	6.71	8.43	38.08	4.95	7.43	49.22
SS70-3d	4.98	48.35	55.51	7.34	13.06	23.26
SS30-Sd-3d	10.66	14.93	52.41	11.41	7.54	28.62
SS30-Sd-7d	6.72	12.06	49.24	7.90	7.90	34.95
SS30-Sd-28d	6.01	19.43	50.17	15.67	8.71	25.08
SS30-HW-3d	5.35	10.21	53.66	5.02	2.70	37.83

## Data Availability

No new data were created or analyzed in this study. Data sharing is not applicable to this article.
